# Attenuated Response of Aged Mice to Respiratory *Francisella novicida* Is Characterized by Reduced Cell Death and Absence of Subsequent Hypercytokinemia

**DOI:** 10.1371/journal.pone.0014088

**Published:** 2010-11-23

**Authors:** Chris A. Mares, Jyotika Sharma, Sandra S. Ojeda, Qun Li, Jocelyn A. Campos, Elizabeth G. Morris, Jacqueline J. Coalson, Judy M. Teale

**Affiliations:** 1 Department of Microbiology and Immunology, The University of Texas Health Science Center at San Antonio, San Antonio, Texas, United States of America; 2 South Texas Center for Emerging Infectious Diseases and Department of Biology, The University of Texas at San Antonio, San Antonio, Texas, United States of America; 3 Department of Pathology, The University of Texas Health Science Center at San Antonio, San Antonio, Texas, United States of America; David Geffen School of Medicine at University of California Los Angeles, United States of America

## Abstract

**Background:**

Pneumonia and pulmonary infections are major causes of mortality among the growing elderly population. Age associated attenuations of various immune parameters, involved with both innate and adaptive responses are collectively known as immune senescence. These changes are likely to be involved with differences in host susceptibility to disease between young and aged individuals.

**Methodology/Principal Findings:**

The objective of this study was to assess potential age related differences in the pulmonary host response in mice to the Gram-negative respiratory pathogen, *Francisella novicida*. We intranasally infected mice with *F. novicida* and compared various immune and pathological parameters of the pulmonary host response in both young and aged mice.

**Conclusions/Significance:**

We observed that 20% of aged mice were able to survive an intranasal challenge with *F. novicida* while all of their younger cohorts died consistently within 4 to 6 days post infection. Further experiments revealed that all of the aged mice tested were initially able to control bacterial replication in the lungs as well as at distal sites of replication compared with young mice. In addition, the small cohort of aged survivors did not progress to a severe sepsis syndrome with hypercytokinemia, as did all of the young adult mice. Finally, a lack of widespread cell death in potential aged survivors coupled with a difference in cell types recruited to sites of infection within the lung confirmed an altered host response to Francisella in aged mice.

## Introduction

Infectious diseases including pneumonia, bacteremia, and influenza remain among the top ten causes of morbidity and mortality among the elderly [1]. As age progresses, the immune system undergoes numerous changes that may affect our susceptibility to infection. Immunosenescence is a term that is often used to describe the general alterations that occur within the immune system. Changes with regard to components of cell mediated immunity and humoral immunity have all been reported to fluctuate with respect to age [2], [3], [4]. Recently, components of the innate immune system have also been reported to undergo modifications with regard to age. Functional variations in myeloid cells, especially macrophages and neutrophils, of the innate immune system have been described in aged individuals [5], [6], [7], [8], [9]. While many studies have been directed at tackling the phenomenon of immunosenescence, relatively fewer experimental studies have focused on specific host-pathogen interactions in the context of aging and in a tissue specific manner [10].

*F. tularensis* is a Gram-negative intracellular coccobacillus that causes tularemia, a life-threatening zoonotic infection of humans. Infection can arise with as few as 10 organisms through the inhalation route, and disease caused by this mode of infection can result in overwhelming sepsis that is associated with the highest rate of mortality in mice [11]. *Francisella tularensis* subsp. *tularensis* and subsp. *holarctica* are extremely virulent pathogens capable of causing disease with very low infectious doses especially via the respiratory route [12]. Low infectious doses coupled with ease of dissemination and a history as a bioweapon have led to the inclusion of Francisella on the CDC's Category A list of potential select agents [13]. Delivery of this pathogen through the pulmonary route can lead to the development of flu-like symptoms and subsequent pneumonia, sepsis, and eventually death if left untreated.

*F. tularensis* subsp. *novicida* (*F. novicida*) has been used as a model strain in the laboratory as it is extremely virulent in mice and can be studied with less risk to the investigator. Recent studies have indicated a delay in the immune response in both Schu S4 and *F. novicida* infections *in vivo* [14], [15]. Our laboratory has shown that young mice infected with *F. novicida* develop an aberrant immune response or hypercytokinemia after the initial delay and also show a profound release and upregulation of two damage associated molecular patterns often linked with the onset of severe sepsis [14]. Other evidence has recently reinforced that the fatal outcome in *F. novicida* infected mice includes the development of hypercytokinemia as well as other characteristics typically associated with the onset of severe sepsis [16], [17].

As mentioned previously, infectious diseases, particularly those involving the lung, continue to have a substantial impact on the quality of life among the elderly [18]. One potential explanation for this may be due to the general immunocompromised state of the elderly [19]; however, relatively less is known about how tissue specific immune defenses may be altered with respect to age. Therefore, the goal of these experiments was to determine potential differences in the pulmonary immune response to *F. novicida* between young and aged mice *in vivo*. Our results highlight several important differences between young and aged infected mice in terms of both the nature and kinetics of the resulting immune response.

## Materials and Methods

### Ethics statement

All the experimental procedures were in compliance with Federal guidelines and were approved by the institutional animal care and use committee (IACUC) at both The University of Texas at San Antonio and The University of Texas Health Science Center at San Antonio.

### Mice

C57BL/6 female mice 6–8 weeks old along with 22–24 month old female mice were purchased from Harlan (Indianapolis, IN). Intranasal infection was performed by initially anesthetizing mice with an intramuscular injection of 100 µl ketamine-xylazine mixture (30 mg/ml ketamine, 4 mg/ml xylazine in phosphate-buffered saline [PBS], diluted 1∶5 in PBS) followed by inoculation of 10 µl of bacterial suspension in each nostril drop by drop (20 µl total volume) and allowing the mice to slowly inhale the inoculum.

### Bacterial strains and culture media

*Francisella novicida* strain was obtained from Dr. Bernard Arulanandam (UTSA) through Dr. Fran Nano (University of Victoria). *F. novicida* was grown in Tripticase Soy Broth (Becton Dickinson, Franklin Lakes, NJ) supplemented with 0.1% cysteine (TSAcys) and plated on Trypticase Soy Agar supplemented with 0.1% cysteine (BD). *F. novicida* was then resuspended and taken to a titer between 4×10^2^ cfu/20 µl and 9×10^2^ cfu/20 µl for various experiments. Actual numbers of viable organisms inoculated were confirmed at the time of infection by re-plating on TSAcys.

### Bronchoalveolar Lavage

At serial time points a tracheotomy was performed after mice were anesthetized with a mixture of ketamine-xylazine; a sterile-flexible cannula attached to a 3 mL syringe was inserted into the trachea. The lungs were lavaged with 0.5 mL aliquots of lavage solution (1X PBS, 3 mM EDTA and 100 µM isoproterenol) up to 3 ml. Bronchoalveolar lavage fluid (BALF) was centrifuged at 1300 rpm for 7 min. The remaining cell pellet was resuspended in sterile PBS and taken to a concentration of 1×10^5^ cells/mL. Cytocentrifugation was performed at 1000 rpm for 7 min followed by Diff-Quik staining (Dade Behring Inc., Newark, DE) for differential cell count.

### Lung frozen section preparation

In order to harvest the lungs, we modified a previously documented protocol to better suit our model [14], [20]. Mice were anesthetized at serial time points with a mixture of ketamine and xylazine as described above. Pericardium and trachea were exposed by dissection. An incision was made in the trachea and a sterile-flexible cannula attached to a 3 mL syringe was inserted. Lungs of mice were inflated slowly with 0.5–0.7 mL of Tissue-Tek OCT compound (Sakura Finetek, Torrance, CA): 2 M Sucrose, 1∶1 (v:v) solution. The trachea as well as the right and left bronchus of each lung was tied off. Inflated lungs were then removed, embedded in Tissue-Tek OCT compound and stored at −80°C. Lungs were sectioned at 9 µm by using Shandon Cryotome SME (Thermo Electron Corporation, Pittsburg, PA). One in every five slides containing lung sections were fixed in formalin for 10 min at room temperature (RT) and stained with hematoxylin and eosin (H&E) to examine lung pathology as well as the degree of cellular infiltration. The remaining slides were air dried overnight and fixed in fresh acetone for 20 s at RT. Acetone fixed sections were wrapped in aluminum foil and stored at −80°C or processed immediately for immunofluorescence microscopy.

### Immunofluorescence microscopy

Lung sections were thawed at RT for 30 min. Terminal deoxynucleotidyl transferase dUTP nick end labeling (TUNEL) staining was then performed in situ using the ApopTag In Situ Detection kit (Chemicon, Billerica, MA) according to the manufacturer's protocol. Sections were then mounted with Fluorsave reagent (Calbiochem, La Jolla, CA) containing 0.6 µM 4′6′ diamidino-2-phenylindole-dilactate-DAPI (Molecular Probes, Eugene, OR). Fluorescence was visualized in a Leica DMR epifluorescent microscope (Leica Microsystems, Wetzlar Germany). Images were acquired using a cooled CCD SPOT RT camera (Diagnostic Instruments Inc., Sterling Heights, MI), and they were processed and analyzed using Adobe Photoshop 7.0 (Adobe, Mountain View, CA) and/or IP Lab 4.0 (Scanalytics, Fairfax, VA).

### Determination of CFUs in tissues and pulmonary host response

Lungs and spleens were collected from euthanized mice that had been previously intranasally infected within a range of 4×10^2^ cfu/20 µL and 9×10^2^ cfu/20 µL. Blood was collected via cardiac puncture with heparin sodium salt used as an anticoagulant. Blood was then serially diluted and the dilutions were plated on TSA plates supplemented with cysteine. Lungs and spleen were aseptically removed and placed in 1 mL of sterile PBS containing a protease inhibitor cocktail (Roche, Indianapolis, IN). Lungs and spleen were then immediately homogenized, and a sample was serially diluted and plated on TSA plates supplemented with cysteine. Plates were grown at 37°C for 24 hrs and CFUs were subsequently calculated. The remainder of the lung homogenate was centrifuged at 10000× g for 10 min at 4°C. The supernatant was collected, aliquoted and frozen at −80°C. A minimum of 300 µL was sent to Rules Based Medicine (Austin, TX) to be analyzed using the Rodent MAP v 2.0 assay in order to assess the level of pulmonary cytokines and other immunological mediators present in the various homogenized samples.

### Statistical Analysis

Survival analysis was generated using the Kaplain-Meyer curve, and statistics were calculated using a log-rank test. Statistics for comparing bacterial burden, cytokine expression, and quantification of pixels was calculated using the Student's *t*-test through SigmaPlot 8.0 and GraphPad Prism 5. Values of *p*<.05 were considered significant.

## Results

### A small proportion of aged mice are capable of surviving an intranasal infection with *F. novicida*

**Figure 1:**
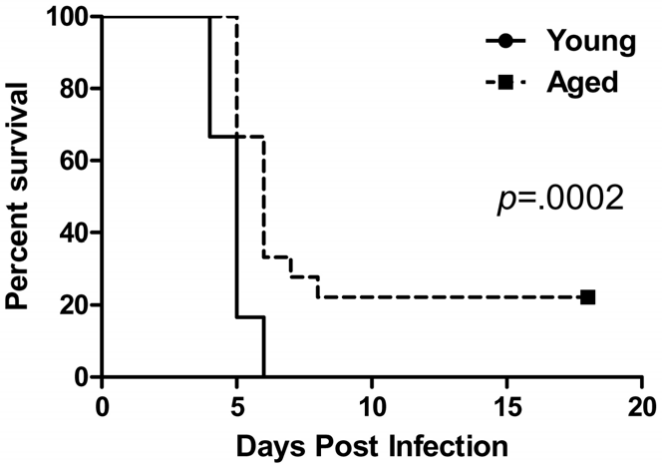
A small proportion of aged mice are able to survive an intranasal challenge with *F. novicida*. Aged mice (22–24 months; *n* = 18) and young mice (8–12 weeks; *n* = 18) were infected intranasally with a dose of 397 CFU/20 µl to 500 CFU/20 µl of *F. novicida* and monitored for survival. Some of the aged mice survived the challenge (4/18) while all of the young mice succumbed to the infection. Data was plotted using a Kaplain-Meier curve and statistical analysis was determined by a log rank test (*p* = .0002).

We intranasally infected both young (8–12 week old) and aged mice (22–24 month old) with *F. novicida* with 4–9×10^2^ CFU/20 µL. In our laboratory a dose of about 3×10^2^ CFU/20 µL is sufficient to cause mortality in 100% of young mice within 4 to 6 days post infection. After infection, we monitored the mice of each age group for the next 16 days (Fig. 1). Initially we noticed that young mice were exhibiting several symptoms including hunched posture, piloerection, lethargy and in severe cases eye discharge beginning on day 3 post infection. These symptoms persisted and worsened in the young animals, and all of them died between 4 and 6 days post infection (*n* = 18). Symptoms in the aged group were difficult to detect at 3 DPI as this age group appeared similar to mock-infected aged mice and were still quite active. However, the symptoms that were evident in the young mice at 3 DPI began to appear at 4 DPI in the infected aged mice, and most aged mice succumbed to infection between days 5 and 6 post infection. However, a small, but significant proportion survived until day 8 post infection and continued to survive or seemingly cleared the infection by day 16 post infection (4/18; *p* = .0002).

### Aged mice display an altered respiratory pathology to *F. novicida* infections

**Figure 2:**
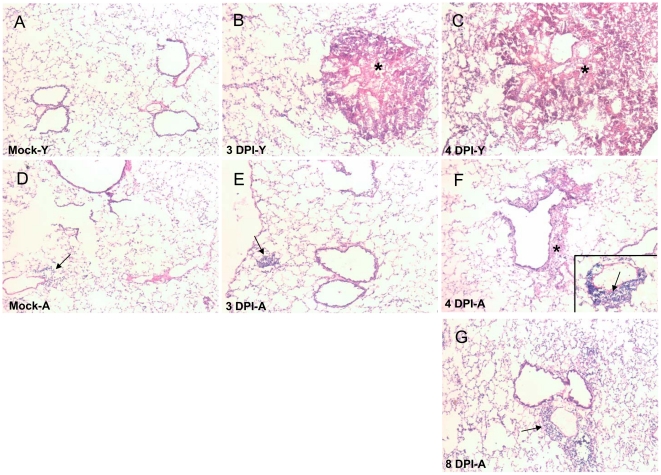
Aged mice display an altered pulmonary pathology in response to *F. novicda*. Young and aged mice were intransally infected with *F. novicida*. Lungs were harvested at various time points and H&E staining was performed to help assess the pathology associated with the course of infection. Panels A and D show mock lungs from young and aged mice, respectively. The arrow indicates perivascular mononuclear cells present in mock aged lung. Panels B and E depict the lungs of young and aged mice at 3 DPI. The asterisk is highlighting large foci of necrosis that is typical by 3 DPI in the lungs of young mice. These foci were notably absent in aged mice. However, small perivascular and peribronchial aggregates of viable mononuclear cells (arrow) were commonly found in the aged lungs. Panels C and F show young and aged lungs at 4 DPI while panel G exemplifies a lung from an aged mouse at 8 DPI. Both C and F are characterized by large foci of necrosis and lung consolidation (asterisk). At 8 DPI (G) in the aged mice there are several perivascular and peribronchial mononuclear aggregates and a few are also present at 4 DPI in moribund aged mice (F, arrow). Magnification 100X.

In mock-infected controls and at the early time points of 6 hr and 1 day post infection the lungs of young and aged mice exhibited minor differences in terms of the cellular constituency (Fig. 2A, D and data not shown). In aged mocks, several mononuclear (monocytic) cells were present in the lungs although they seemed to be mainly present in perivascular and peribronchial areas (Fig. 2D). This cell type was generally absent from the lungs of mock young mice. Increases in the size and in some cases frequency of these perivascular infiltrates were observed at 6 hrs post infection and also at 1 DPI in aged mice (data not shown). Such aggregates were notably absent in young mice through the first two days of infection. However, by 3 DPI, the lungs of young mice began to display large foci of necrotic infiltrates and tissue destruction (Fig. 2B). Fewer and smaller foci were detected in their aged counterparts at the same time point (Fig. 2E). However, at 4 DPI (Fig. 2F) the lungs of some aged mice did begin to present with large necrotic infiltrates as seen in their young counterparts at 3 DPI (Fig. 2B). The lungs of young mice at 4 DPI showed a high level of confluence with an increasing area of necrotic tissue occluding the normal architecture of the lung (Fig. 2C, asterisk). We were not able to harvest the lungs from young mice past day 4 post infection due to the death of the animals but we were able to harvest lungs from aged mice at days 6 and 8 post infection. Surprisingly, in a subset of aged mice that survived the infection, a substantial increase in mononuclear infiltrates was evident in their lungs (Fig. 2G, arrow). However, in contrast to the infiltrates observed in moribund young or aged mice, these cellular aggregates appeared to be comprised of viable cells and were presumably protective.

### Aged mice initially have lower bacterial burdens than young mice

**Figure 3:**
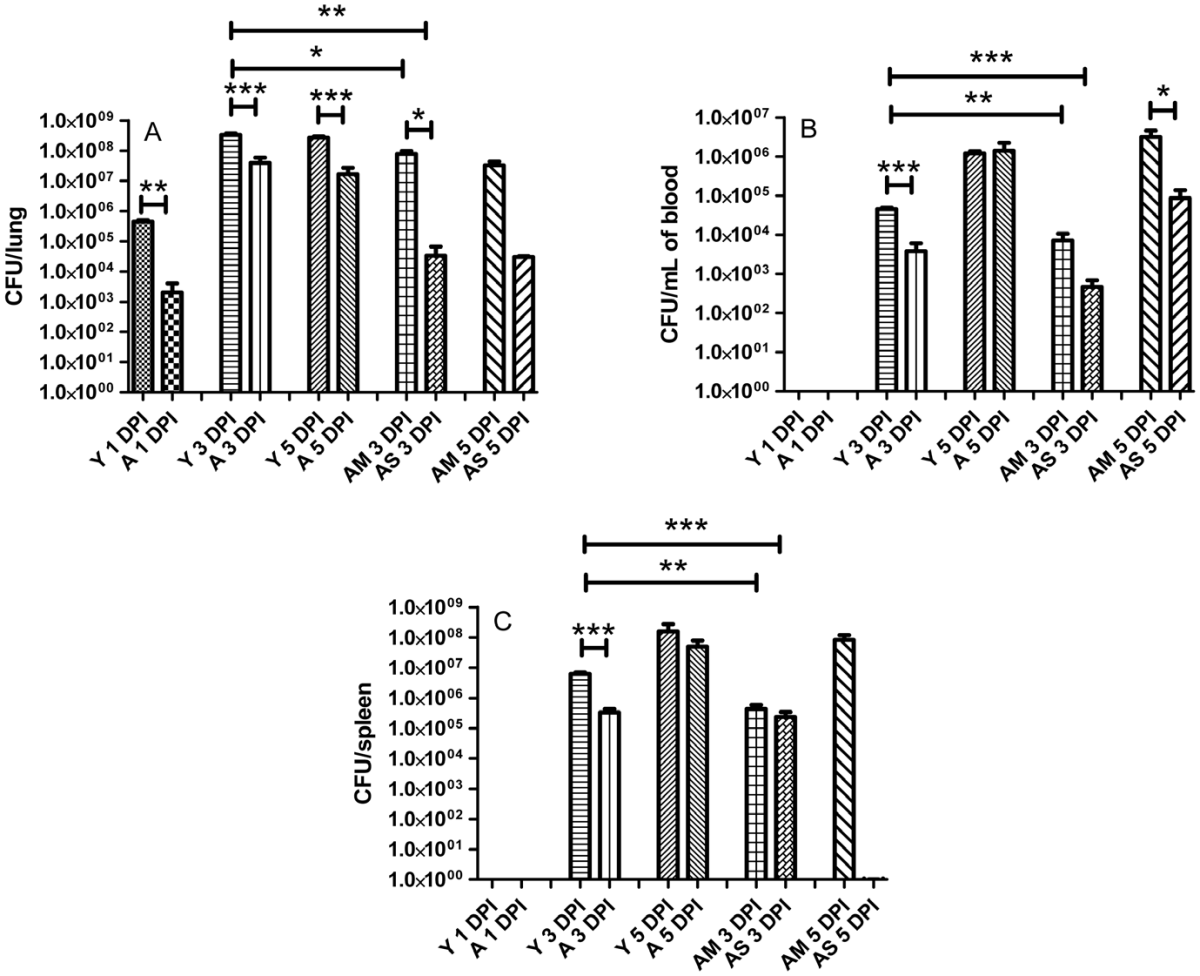
Bacterial burdens in aged mice are reduced early in the infection. Young and aged mice were intranasally infected and their lungs, spleen and blood were harvested, processed, and plated in order to determine the bacterial burden (*n* = 3–4 mice per group). Panel A shows a significant reduction in the number of bacteria recovered from the lungs of aged mice as compared to young mice throughout the course of infection, especially at 1 and 3 DPI. Panel B indicates a reduction in systemic dissemination in the blood between young mice and aged mice that could be considered potentially moribund and aged mice that seemed to be able to control the infection. Panel C shows the bacterial burden in the spleen and similarly indicates a reduction in the amount bacteria recovered from aged mice (survivors and non-survivors) relative to young mice. * *p*<.05; **p<.005; ***p<.001.

We then compared the bacterial burdens in the lungs, spleens, and blood of the infected young and aged mice. Our results indicated that at 1 DPI, fewer bacteria were detected in the lungs of aged mice as compared to the young (2.0×10^3^ CFU/organ vs. 1.5×10^6^ CFU/organ, respectively *p*<.005). At this initial time point, we were unable to detect systemic spread of the bacteria with this dose. By 3 DPI young mice had significantly higher bacterial loads in the lung, spleen and blood when compared to their aged counterparts. At this time point, aged mice could also be artificially segregated into two groups based on their bacterial loads. One group, which will be called the aged slow progressors (aged survivors, AS), had significantly lower burdens in the lung, blood and spleen than the young (Fig. 3A, B, C; *p*<.005, *p*<.001, *p*<.001, respectively). This group also had a significantly lower bacterial burden in the lung (Fig. 3A, *p*<.05) than the other aged group that will be termed the aged normal progressors (aged moribund, AM) from this point forward. The aged normal progressors (aged moribund, AM) collectively had significantly lower burdens at 3 DPI in their lung, spleen, and blood (*p*<.05, *p*<.005, *p*<.005, respectively). At 5 DPI the aged mice had significantly lower burdens in their lungs (*p*<.005); however, bacterial dissemination to the blood and spleen was relatively similar in both moribund age groups.

### Aged mice have a delayed initial neutrophil response to *F. novicida*

As neutrophils are one of the first cells to respond to an infection we next compared the influx of neutrophils in the bronchoalveolar lavage fluid (BALF) between young and aged mice at early times post-infection. Initially we noticed a slightly elevated percentage of neutrophils in the aged mocks as well as at 6 hours post infection. A significant increase in the percentage of neutrophils was evident at 1 DPI in young mice (*p*<.05) yet this increase was not detected in the aged until day 3 post infection (Table 1). Similarly, total number of neutrophils began to increase at 1 DPI in the young and was significantly higher at 3 DPI when compared to aged at 3 DPI (Table 1, *p*<.005). In fact, the total number of neutrophils present in the BALF remained relatively similar among aged animals through 3 days post infection.

**Table 1:** Kinetics of PMN influx into the BALF in young and aged mice infected with *F. novicida*.

		Mock	6 HPI	1 DPI	3 DPI
**% PMNs in BALF**					
	**Young**	**1.2%+/−0.6%**	**1.3%+/−0.6%**	26.5%+/−4.7% *	**26.1%+/−2.9%**
	**Aged**	**7.7%+/−6.1%**	**5.6%+/−.8%**	7.0%+/−1.7% *	**26.9%+/−5.7%**
**Total PMNs in BALF**					
**(Cells/mL)**					
	**Young**	**7.5×10^3^+/−4.9×10^3^**	**5.0×10^3^+/−2.0×10^3^**	**2.1×10^5^+/−7.9×10^4^**	8.6×10^5^+/−1.4X10^5 **^
	**Aged**	**2.6×10^4^+/−2.2×10^4^**	**1.7×10^4^+/−7.7×10^3^**	**1.3×10^4^+/−6.2×10^3^**	6.7×10^4^+/−3.5×10^4 **^

BALF was obtained at the indicted times points post infection, cytocentrifuged onto slides and stained with DifQuik to determine the total number and proportion of neutrophils.

Statistics were determined when comparing young and aged samples at individual time points using the Student's t test (* *p*<.05; **p<.005).

**Figure 4:**
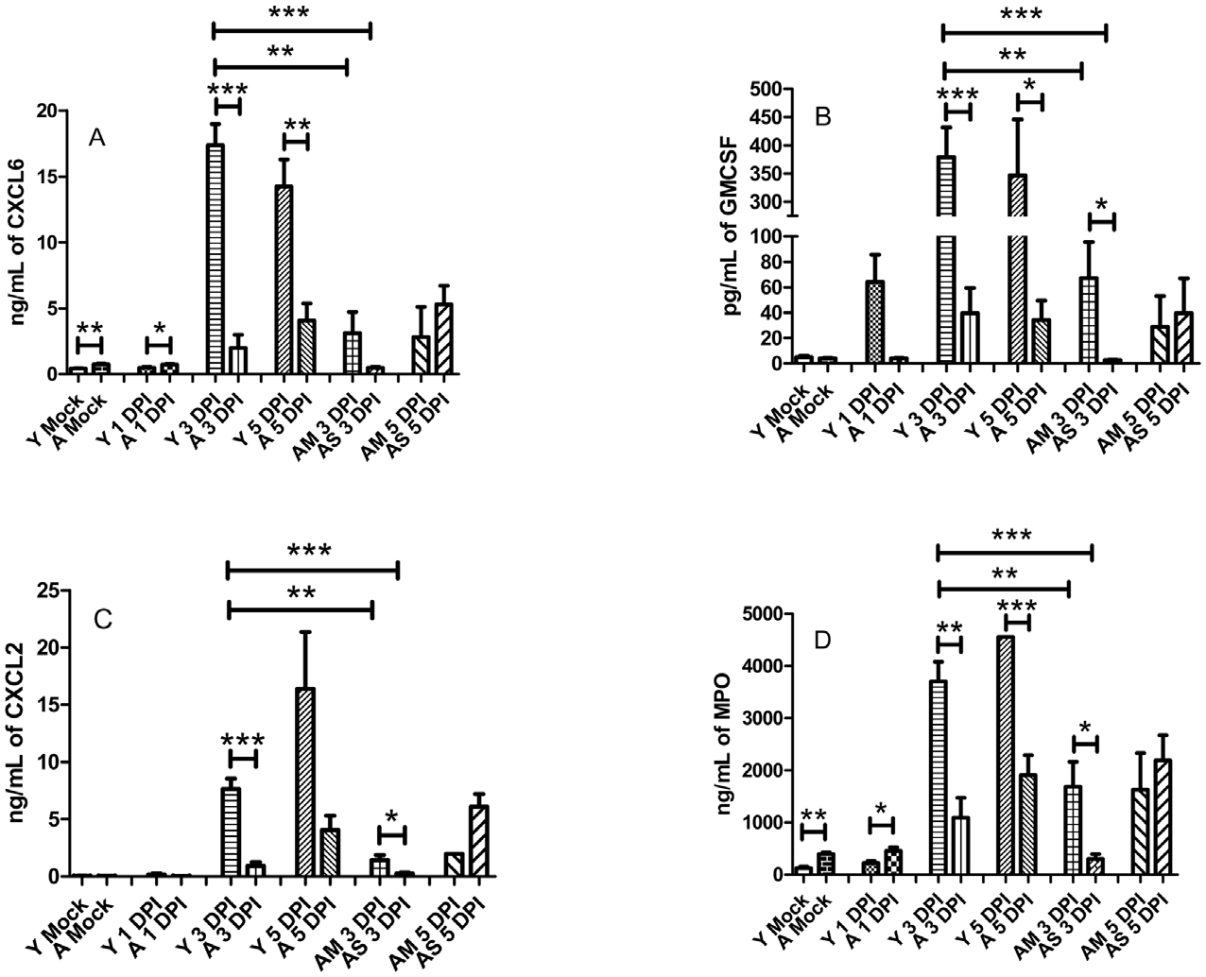
Mediators associated with neutrophil chemotaxis and function are sharply reduced in aged mice. Lungs were aseptically removed from young and aged mice (*n* = 3 to 4 mice per group) and processed to yield whole lung homogenates as described in the material and methods section. The homogenates were then analyzed using the Rodent MAP 2.0 from Rules Based Medicine (Austin, TX). CXCL6 (A), GM-CSF (B), and CXCL2 (C) are all cytokines important for neutrophil chemotaxis and were all less abundant in the lungs of both aged moribund mice and aged survivors. Myeoloperoxidase (D) was also determined to be reduced in the aged, especially in survivors at 3 DPI. *p<.05, **p<.005; ***p<.001.

Due to the difference noted in the kinetics of neutrophil influx we also examined the pulmonary levels of key chemotactic factors in whole lung homogenates. Initially CXCL6 (Fig. 4A, *p*<.005) as well as myeloperoxidase (Fig. 4D, *p*<.005) were found to be at significantly higher levels in the lungs of uninfected aged mice. Both of these mediators remained significantly elevated at 1 DPI in the aged as well (*p*<.05). However, by 3 DPI the levels of CXCL6, CXCL1, GM-CSF, and myeloperoxidase were all significantly increased in the young animals above young mock levels by several fold and were also significantly higher than both the aged slow progressors (aged survivors, AS) as well as the aged normal progressors (aged moribund, AM; Fig. 4). The results also indicate that by 5 DPI the young mice still have significantly higher levels of all of these mediators than their aged counter parts.

### The onset of hypercytokinemia associated with severe sepsis appears to be delayed and in some cases attenuated in aged mice

**Figure 5:**
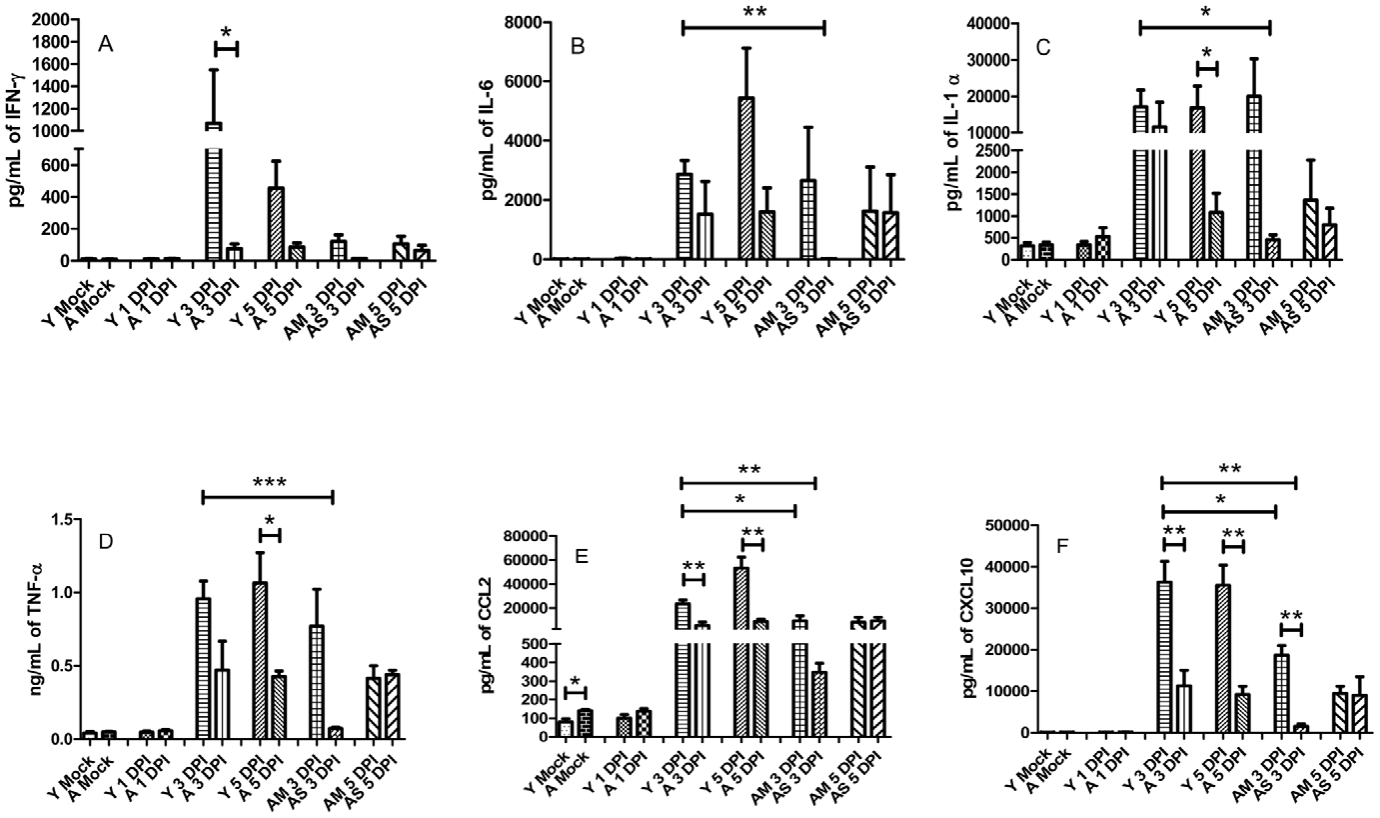
Inflammatory cytokines associated with severe sepsis and hypercytokinemia are at lower levels in aged survivors. Levels of inflammatory cytokines associated with severe sepsis were measured in the lungs of young and mice intranasally infected with *F. novicida* (*n* = 3 or 4 mice per group). Levels of IFN-gamma, IL-6, IL-1 alpha, TNF-alpha, CCL2 and CXCL10 were all elevated at 3 DPI in the young and remained significantly higher than their aged counterparts through 5 DPI. Strikingly, aged survivors had significantly reduced levels in most of these cytokines. * *p*<.05; **p<.005; *p*<.001.

Hypercytokinemia has been associated with a fatal outcome in various infectious and non-infectious models, including this particular model [14], [17]. We therefore wanted to investigate the pulmonary levels of other cytokines in response to this infection. In particular we assessed the levels of pro-inflammatory cytokines including IFN-gamma, IL-6, IL-1 alpha, TNF-alpha, CCL2, and CXCL10 (Fig. 5A–F). We noticed that most of these cytokines were significantly upregulated in the young starting at 3 days post infection. These cytokines were also highly upregulated in the subset of aged animals that appeared to be normal progressors (AM). However, in the aged slow progressors (AS) we noted that the upregulation of these cytokines was abrogated. All of these cytokines remained significantly elevated, in most cases, at 5 days post infection in the young when compared to their aged counterparts at the same time point (Fig. 5A–F).

**Figure 6:**
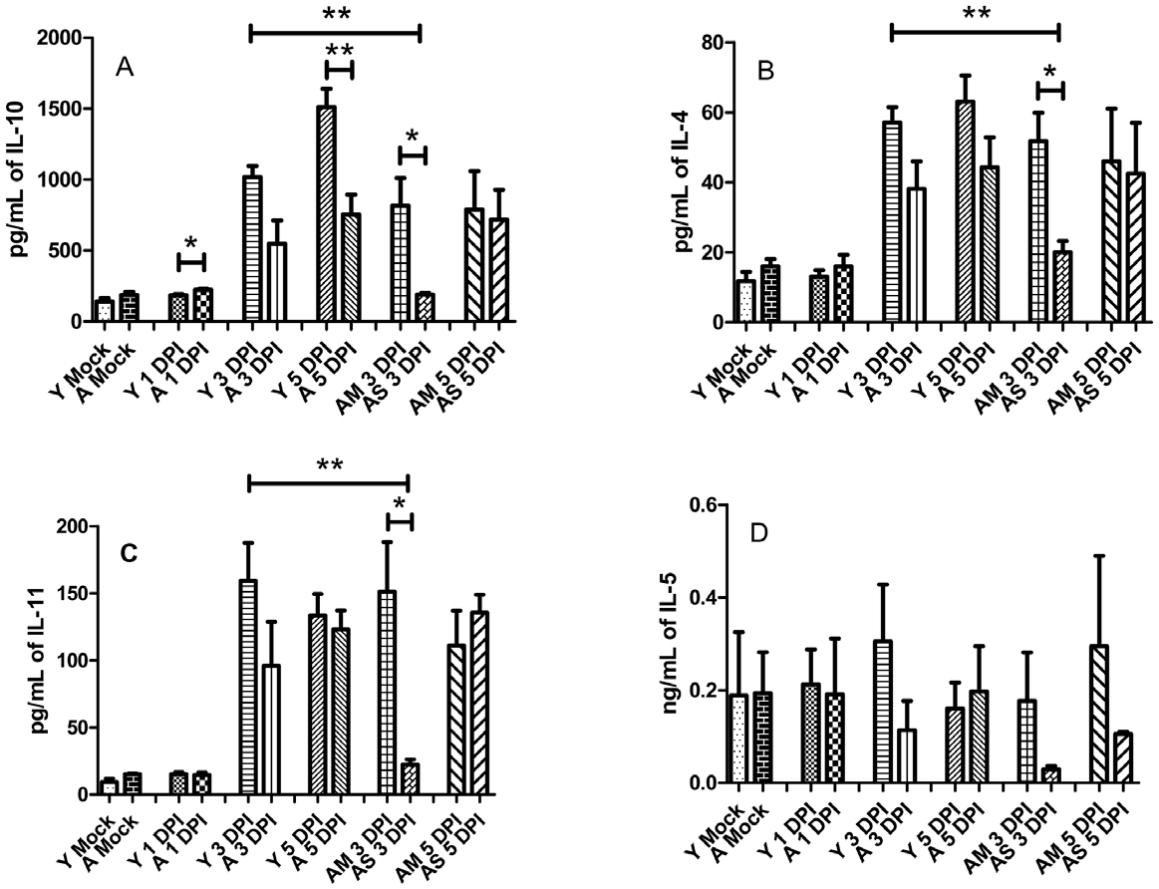
Anti-inflammatory cytokines are also aberrantly upregulated in young mice but not in aged survivors. The levels of anti-inflammatory cytokines were determined in the lungs of young and aged mice infected with *F. novicida* at 1, 3, and 5 days post infection (*n* = 3 or 4 mice per group). IL-10, IL-4, IL-11 and IL-5 (Panels A, B, C and D, respectively) were all determined to be upregulated to various degrees in young mice by 3 DPI. Aged survivors presented with significantly lower levels of IL-10, IL-4, and IL-11 at 3 DPI. * *p*<.05, ** *p*<.005, *** *p*<.001.

As anti-inflammatory cytokines can also play a role in the response to severe sepsis we next looked at the levels of IL-10, IL-4, IL-11 and IL-5 [21]. A similar pattern was observed with the anti-inflammatory markers as was observed with the pro-inflammatory mediators. IL-10, IL-4 and IL-11 were all upregulated at 3 DPI in young mice and aged normal progressors (AM) (Fig. 6A, B, and C). Again, in aged slow progressors (AS) the levels of these cytokines were drastically reduced.

### Cell death and tissue destruction is less pronounced in the lungs of aged mice

**Figure 7:**
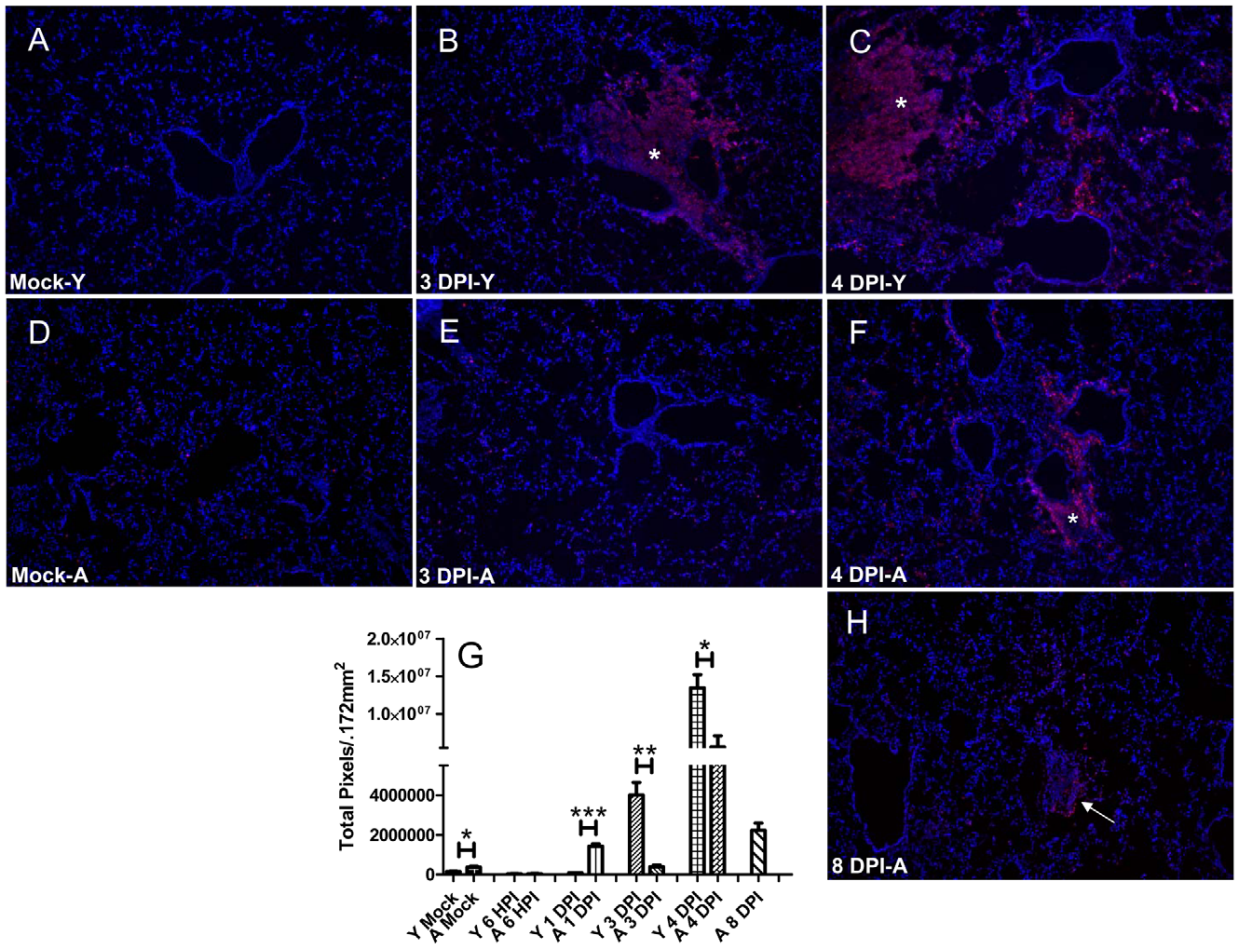
Extent of cell death correlates with outcome of respiratory tularemia in young and aged mice. Young and aged mice were intranasally infected with *F. novicida* and their lungs were subsequently harvested and processed for in situ fluorescent TUNEL staining. In situ TUNEL staining was performed according to manufacturer's protocol with slight modifications. Panels A and D show TUNEL staining from mock lungs in young (Y) and aged (A) mice. Panels B and E are representative micrographs of young and aged mice at 3 DPI. Note the large necrotic infiltrate present at 3 DPI in the lungs of young mice (asterisk). Panels C and F are illustrating young and aged mice at 4 DPI, respectively. Lung consolidation and necrosis was present throughout the tissue in young mice as indicated by TUNEL (asterisk). Aged mice that appeared moribund at 4 DPI also showed a sharp increase in the extent of TUNEL positive areas (asterisk in F). Panel H represents a lung from an aged survivor at 8 DPI. The arrow indicates a perivascular aggregate. Strikingly this area, as well as the entire lung contains less TUNEL positive cells than moribund young and aged mice. Panel G is the quantification of TUNEL staining in a representative of 3 independent experiments. A significant increase in TUNEL was observed at 3 DPI and 4 DPI in the young mice as well as at 1 DPI and at 4 DPI in aged mice (*n* = 3 per group except 8 DPI where *n* = 2).

Unresolved cell death with secondary necrosis has also been associated with severe sepsis [22]. In order to assess the level of cell death in our model we used an in situ TUNEL staining to visualize the extent of cell death throughout the course of infection. In young mice we noticed a remarkable increase in TUNEL positive areas at 3 and 4 days post infection (Fig. 7B and C). The TUNEL positive areas seemed to localize around bronchioles and blood vessels and both the intensity and amount of cells positive for TUNEL increased with time post infection. The aged mice did not show a robust increase in TUNEL staining until 4 DPI. The only time point post infection where aged mice had more TUNEL positive staining was at 1 DPI, albeit at considerably lower levels than observed in moribund mice. Interestingly, the pattern at 3 DPI appeared to be limited to individual cells or small infiltrates in contrast to the widespread cell death phenomenon observed in young mice at 3 DPI (Fig. 7B and E). However at 4 DPI, the aged mice that appeared moribund appeared very similar to young mice that were taken at 3 and 4 DPI (Fig. 7B, C and F). These mice showed an increased level of TUNEL staining over mock levels; however, they did not seem to approach the levels observed in the moribund young or aged mice at 3 and 4 DPI (Fig. 7B and C). At 8 DPI (Fig. 7H), the amount of TUNEL staining was elevated above mock levels but was remarkably lower when compared to moribund mice at 3 and 4 DPI (Fig. 7B and C) and the expression seemed to be limited to individual aggregates of cells dispersed throughout the tissue.

## Discussion

Aging has been associated with a change in immune function. These alterations have been described as the potential basis for the increased susceptibility to pathogens and the diseases they cause. Direct evidence ascertaining the relationship between age and infection with Francisella in humans is difficult to interpret as some studies indicated a positive correlation between Francisella infection and age and others a negative one [23], [24], [25]. An important caveat to the epidemiological studies is that they usually combine several different routes of infection. Studies from our lab have indicated that the route of infection with Francisella is an important determinant of bacterial dissemination as well as disease progression and outcome [26].

Our data indicate that the aged response is altered to Francisella and the changes seen in a small proportion of aged mice result in survival to a lethal intranasal challenge with *Francisella novicida*. The survival studies in conjunction with the difference in pathology between aged and young mice verified the presence of age related differences in the host response to Francisella. We also found a delayed response with regard to neutrophil influx into the site of infection in aged mice as compared to young. We speculate that this may be due to the altered cytokine milieu in the uninfected aged lung as well as the rapid appearance of perivascular infiltrates at 6 hrs and 1 DPI that was lacking in young animals. Surprisingly, aged mice were more adept at controlling the growth of *F. novicida* in the pulmonary compartment throughout 5 days PI. Nevertheless, despite the ability of aged mice to control bacterial burdens in the lungs, the majority of them succumb to infection. One possibility could be that although the pulmonary response is better in terms of controlling the bacterial burden in aged mice throughout the course of infection, the systemic dissemination of *F. novicida* ultimately reached similar levels in young mice and aged mice (especially aged moribund) at later times post infection. Interestingly, we noted significantly elevated levels, albeit relatively low, of myeloperoxidase, CXCL6, CCL2, and MMP-9 (data not shown) levels in mock aged mice compared with mock young controls. This suggests an already existing level of inflammation that could potentially be playing a role in helping to control the bacterial burdens early in aged mice. Future interest will be focused on discovering the underlying mechanism associated with aged survivors.

As mentioned before, other models as well as clinical and epidemiological studies have indicated that advanced age can be associated with higher mortality and morbidity [27], [28], [29]. However, a few studies have shown that aged mice may be better able to cope with intracellular infections in particular. For example, Cooper et al. [30] have reported previously that aged mice were able to initially inhibit replication of *Mycobacterium tuberculosis* growth in the lungs appreciably more than young mice. Similar studies by Turner et al. [31] confirmed that growth of *M. tuberculosis* was significantly attenuated early in aged mice. Studies conducted by High et al. [32] have also recently suggested that aged mice have a more favorable outcome when dealing with *Brucella abortus* with respect to bacterial burdens at later times post infection. Ehrchen et al. [33] also reported that senescent BALB/c mice experienced less footpad swelling and a reduced *Leishmania* parasite load in the spleen. Furthermore, recent studies from our laboratory using a less virulent strain of Francisella (LVS) have also led to similar findings described in the current study in terms of pathology and an altered pulmonary response in aged mice [34]. As all of these organisms including Francisella replicate intracellularly, the results suggest an enhanced response in aged mice to intracellular pathogens of macrophages. It is also possible that the lower bacterial burdens associated with the aged response to *F. novicida* could be impacting the kinetics and may correspond directly with the lower expression of cytokines in the lungs of aged mice, especially those aged mice which appear to be slow progressors or potential survivors (AS). However, it is worth reiterating that bacterial burdens did confirm that even potential survivors harbored a substantial amount of Francisella in their lungs and spleens at 3 DPI (∼10^5^ CFU/organ) as well as blood. Moreover, such levels in young adult mice consistently lead to death in a few days.

Macrophages have been reported to undergo numerous changes throughout the aging process [35]. Some of these changes include alterations in phagocytosis and a reduction in cytokine production [8]. While the experiments described in this study did not directly focus on the role of the aged macrophage in response to Francisella infections, many of the observations with regard to the attenuated cytokine production and the delay in the chemotaxis of neutrophils to the site of infection were consistent with previously reported studies that have focused on changes in macrophage function [36]. Exploring the potential role of the aging macrophage in response to Francisella infections is a current area of investigation in our laboratory.

The onset of cell death and subsequent hypercytokinemia are both hallmarks of the acute onset of severe sepsis associated with Francisella infections [14], [17], [37], [38]. Normally in young mice infected with Francisella, cell death is widespread initially at 3 DPI and continues to worsen with time post infection until death. TUNEL staining in these mice appears confluent, especially around blood vessels and highlighting large foci of necrotic infiltrates that have been shown to be rich sources of damage associated molecular patterns (DAMPs) that can potentially further exacerbate host mediated tissue destruction [14], [17]. However, one observation we made in the aged mice was an increase in TUNEL staining initially in aged mice at 1 DPI. Notably, the pattern at this time point is confined to individual aggregates or cells in the lung. It could be hypothesized that the initial increase in apoptotic cells could be a more controlled response that allows for the apoptotic debris to be taken up more efficiently. We also found that both pro and anti-inflammatory cytokines were highly upregulated at 3 DPI and 5 DPI in young mice as is consistent with hypercytokinemia associated with severe sepsis [39]. However, the response in aged mice appeared to be blunted. At day 3 post infection, mice could be separated based on their bacterial burdens and pulmonary cytokine expression into either slow progressors or potential aged survivors and those which were more likely to succumb to infection within the normal time frame. Cell death and hypercytokinemia were strikingly absent or reduced in mice that were deemed potential survivors. Further studies are necessary to dissect immune parameters associated with survival.

In summary aged mice displayed an attenuated response to respiratory *F. novicida* infections. This included a reduction in lung bacterial burden early in the infection. In general, aged mice also displayed a less vigorous pulmonary cytokine response with aged survivors showing an even further reduction in production of pulmonary cytokines. Studies are ongoing to determine potential differences in kinetics, distribution and function of distinct leukocyte subsets in infected lungs of young and aged mice.
